# Genetic Variability of West Nile Virus in US Blood Donors, 2002–2005

**DOI:** 10.3201/eid1403.070463

**Published:** 2008-03

**Authors:** Andriyan Grinev, Sylvester Daniel, Susan Stramer, Susan Rossmann, Sally Caglioti, Maria Rios

**Affiliations:** * Food and Drug Administration, Bethesda, Maryland, USA; † American Red Cross, Gaithersburg, Maryland, USA; ‡ Gulf Coast Regional Blood Center, Houston, Texas, USA; § Blood System Laboratories, Tempe, Arizona, USA

**Keywords:** West Nile virus, virus evolution, United States, genetic variation, phylogenetic analysis, flavivirus, molecular epidemiology, research

## Abstract

West Nile virus (WNV) was detected in the United States in 1999, has reoccurred every summer since, and has become endemic. Transfusion transmission was documented in 2002, and screening of blood donations for WNV began in 2003. We investigated genetic variation of WNV in human isolates obtained from specimens collected from 30 infected blood donors who tested positive for WNV RNA during 2002–2005. Complete genomic sequences of 8 isolates and structural gene sequences from 22 additional isolates were analyzed. We found some genetic diversity in isolates from different geographic regions and genetic divergence from reported sequences from epidemics in 1999–2001. Nucleotide divergence of structural genes showed a small increase from 2002 (0.18%) to 2005 (0.37%), suggesting absence of strong selective pressure and limited genetic evolution of WNV during that period. Nevertheless, WNV has continued to diverge from precursor isolates as geographic distribution of the virus has expanded.

## Abstract 2

This virus is diverging from precursor isolates as its geographic distribution expands.

*West Nile virus* (WNV) is a small, enveloped, positive-strand RNA virus of the genus *Flavivirus* and a member of the Japanese encephalitis serocomplex. The plus-sense RNA genome is ≈11 kb and contains a single open reading frame flanked by 5′ and 3′ untranslated regions (UTRs). The encoded polyprotein is processed into 3 structural and 7 nonstructural (NS) proteins that are essential for viral replication, assembly, and release. WNV is maintained in nature by transmission between mosquitoes and birds but can also infect humans and other mammals (*1*) and reptiles (*2*). Most human infections are asymptomatic (70%–80%); symptomatic cases range from flulike illness to severe neurologic disease (>1% of cases) (*3*–*5*).

The first US outbreak of WNV occurred in 1999 in New York City; 68 human infections, mostly as meningoencephalitis, were confirmed, resulting in 7 deaths. Since 1999, WNV epidemics have reoccurred yearly with 23,975 reported human cases of disease and 962 deaths through 2006 (www.cdc.gov/ncidod/dvbid/westnile).

In 2002, the virus spread westward and the number of reported human cases increased dramatically. The North American epidemics of 2002 and 2003 represent the largest WNV outbreaks ever reported (*6*). Additional modes of WNV transmission were identified in 2002, including human-to-human transmissions from mother to child, organ transplantation, and blood transfusion (*7*–*9*). The quick spread of the virus raised questions regarding viral adaptation and prompted a detailed investigation of the genetic evolution of the virus.

The prototype WNV isolate, NY99-flamingo382–99 (WN-NY99; GenBank accession no. AF196835) was obtained from a flamingo infected during the 1999 outbreak in New York. This isolate belongs to lineage I and is most closely related to the Israel-98 goose isolate AF481864 (IS-98) (*6*,*10*). Phylogenetic comparisons of partial and complete nucleotide sequences from US isolates collected between the 1999 and 2000 epidemics with WN-NY99 isolate showed a high degree of genetic similarity with >99.8% nucleotide homology and >99.9% amino acid homology (*11*–*14*). A study of 22 different WNV isolates from 2001 and 2002 showed genetic variation of 0.35% (mean 0.18%) in the premembrane (*preM*) and envelope (*env*) genes compared with WN-NY99 (*15*).

Subsequently, 2 distinct genotypes were detected in strains obtained from Texas in 2002 (*15*). One new genetic variant widely spread over the United States diverged from the original WN-NY99 strain by several conserved nucleotide mutations and 1 aa substitution in the env protein. Recent studies have highlighted the emergence of a new WNV dominant genotype, named WN02, which has been increasingly prevalent in the United States since 2002 (*16*–*19*). Although 13 nt mutations became fixed in the new dominant genotype compared with the WN-NY99 prototype, the highest nucleotide sequence divergence of WNV strains isolated after 2002 is still in the range of 0.4%–0.5% (*18*,*20*). The reason for displacement of the WN-NY99 genotype by a new dominant genotype in North America is not clear, but could have been caused by differences in transmission efficiency of domestic mosquitoes that may offer a selective advantage for the newly emerged genotype (*20*,*21*).

Several studies on the genetic variation of WNV have been published (*12*,*15*,*17*,*19*,*21*,*22*). However, continuous monitoring of variability is needed because sensitivity of blood donor screening and diagnostic assays may be affected, producing a negative effect on public health. Genetic variability could also affect viral pathogenesis, development of vaccines, and development of efficacious therapeutic agents.

This study reports the genomic variation of WNV observed in clinical isolates obtained in the continental United States during 4 consecutive years (2002–2005). We observed an increase in the number of mutations in the full WNV genome from 0.18% in 2002 to 0.37% in 2005. It should be noted that 80% of the nucleotide changes in structural regions are transitions (T ↔ C) and 75% are silent mutations. Thus, WNV has continued to slowly diverge from precursor isolates as geographic distribution of the virus expanded.

## Materials and Methods

### Study Samples

This study included 30 plasma specimens (Table 1) obtained from blood donor units positive for WNV by nucleic acid tests used to screen blood donations under Food and Drug Administration (FDA)–approved nationwide clinical trials; the specimens were collected in 13 states in the continental United States. All samples were collected under Institutional Review Board–approved informed consent provided by each of the institutions performing donor screening.

**Table 1:** Sequences of 30 West Nile virus isolates from the United States*

Isolate ID	Year	Geographic location	No. passages in Vero cells†	GenBank accession no.
**FDA/HU-02**	**2002**	**NY**	**3**	**AY646354**
**ARC10–02**	**2002**	**MI**	**1**	**AY795965**
ARC12–02	2002	OH	1	DQ666453
ARC13–02	2002	MI	1	DQ666454
ARC15–02	2002	MI	1	DQ666455
ARC16–02	2002	IN	1	DQ666456
ARC17–02	2002	GA	1	DQ666457
**BSL5–03**	**2003**	**UT**	**1**	**DQ005530**
BSL9–03	2003	TX	1	DQ666458
BSL56–03	2003	ND	1	DQ666459
BSL62–03	2003	SD	2	DQ666460
BSL114–03	2003	TX	2	DQ666461
RMS1–03	2003	MN	1	DQ666462
RMS2–03	2003	IN	1	DQ666463
RMS3–03	2003	IN	1	DQ666464
RMS4–03	2003	IA	1	DQ666465
BSL1–04	2004	AZ	1	DQ666466
BSL2–04	2004	AZ	1	DQ666467
BSL4–04	2004	AZ	2	DQ666468
**BSL5–04**	**2004**	**AZ**	**1**	**DQ666448**
BSL6–04	2004	AZ	1	DQ666469
BSL7–04	2004	AZ	2	DQ666470
BSL8–04	2004	AZ	2	DQ666471
**GCTX1**	**2005**	**TX**	**1**	**DQ666449**
**GCTX2**	**2005**	**TX**	**1**	**DQ666450**
**BSL2–05**	**2005**	**SD**	**1**	**DQ666452**
BSL6–05	2005	AZ	1	DQ666472
BSL9–05	2005	TX	1	DQ666473
BSL10–05	2005	LA	1	DQ666474
**BSL13–05**	**2005**	**AZ**	**1**	**DQ666451**

*Isolates in **boldface** have been completely sequenced. †Indicates passage of viral isolate from which RNA extracts were used to obtain the genomic sequence.

### Virus Isolation in Vero Cells

Vero cells were plated in T75 flasks and grown to 85% confluence in Eagle minimal essential medium (GIBCO BRL, Gaithersburg, MD, USA) containing 5% fetal bovine serum (Hyclone, Logan, UT, USA) and 10 μg/mL of penicillin/streptomycin (GIBCO-BRL). For viral isolation, medium was removed, and 500 μL of each plasma sample was added to individual flasks and the volume was adjusted to 5 mL with fresh medium. Cultures were incubated for 2 hours with the plasma, either at room temperature with gentle rocking or at 37°C with sporadic mixing. A total of 10 mL of fresh medium was then added and cultures were incubated at 37°C in an atmosphere of 5% CO_2_. Cultures were observed daily for cytopathic effect under phase microscopy. Supernatants were harvested when an extensive cytopathic effect was observed, centrifuged to remove cell debris, and frozen at –70°C until further analysis.

### RNA Extraction, Reverse Transcription, and PCR

RNA extracts were obtained from 1-mL plasma samples by using Trizol reagent (Invitrogen, Carlsbad, CA, USA), and extracts were resuspended in 20 μL of water. RNA samples from viral passages were isolated from 140 μL of culture supernatants by using the QiaAMP viral RNA extraction kit (QIAGEN, Valencia, CA, USA) according to the manufacturer’s protocol. RNA extracts were stored at –70°C until further analysis. Reverse transcription reactions were performed in a final volume of 20 µL that contained 10 μL of RNA and specific WNV reverse primers by using SuperScript III (Invitrogen) according to the manufacturer’s instructions. Specific primers used for both PCR amplification and sequencing were designed according to available sequence information in GenBank and based on alignments of published WNV sequences. PCR amplification of overlapping fragments to cover the complete viral genome was performed by using 15 pairs of primers (Table 2). Partial sequences for the structural region were obtained by using primer sets 1, 2, and 3. cDNA specimens were amplified by using the Hi-Fidelity PCR system (Invitrogen) according to the manufacturer’s instructions.

**Table 2:** Primer sets used for PCR analyses of West Nile virus and sizes of overlapping amplicons*

Set/location	Amplicon size, kb	Name	Sequence (5′ → 3′)
1/F1–R1300	1.3	F1	AGTAGTTCGCCTGTGTGAGCTGAC
		R1300	TTGGCGCATGTGTCAATGCT
2/F980–R2000	1.0	F980	CTTGGAATGAGCAACAGAGA
		R2000	GTTAGGTCGTTCAATGAAGC
3/F1690–R2685	1.0	F1690	GAGACGTTAATGGAGTTTGA
		R2670	CTTCACTGCTTCCCACATTTG
4/F2340–R3420	1.1	F2340	TTCGGAGGCATGTCCTGGAT
		R3420	CTGATCTCCATACCATACCAACA
5/F3330–R4120	0.8	F3330	GAGAGCTGCGGACACCGTGGACC
		R4120	CATAGCAGACTTGCTCCTTTCT
6/F4070–R4950	0.9	F4070	CTGTTGATGGTCGGAATAGG
		R4950	CCTGGTTTCGTCTGGACGTT
7/F4810–R5650	0.85	F4810	CGCCTGGACCCATACTGG
		R5650	CCATTCGTATCCAGAGTTCCA
8/F5510–R6430	0.9	F5510	AGCATTGCAGCAAGAGGTTA
		R6430	TAGTGCCTGGTGATCCGAGTACAC
9/F6290–R6770	0.5	F6290	CGACCGGAGGTGGTGCTTTGATGG
		R6770	CCTGGAACTTCAGCCATCCA
10/F6690–R7550	0.85	F6690	CCTCCTCATGCAGCGGAA
		R7550	GAGCTTGCTCCATTCTCCCA
11/F7420–R8260	0.85	F7420	CCACACCCATCATGCAGAA
		R8260	CGTTGGAGCAGCTCCATCTT
12/F8170–R9050	0.9	F8170	CATAGGACGATTCGGGTCCT
		R9050	CTCTTTCCCATCATGTTGTAAATGC
13/F8920–R9810	0.9	F8920	CAGCTTTGGGTGCCATGTT
		R9810	GAACCTGCTGCCAATCATACC
14/F9750–R10630	0.9	F9750	TCCTCAATGCTATGTCAAAGGT
		R10630	GGTCCTCCTTCCGAGACGGT
15/F10550–R11029	0.5	F10550	TGAGTAGACGGTGCTGCCTG
		R11029	GATCCTGTGTTCTCGCACCACCAG

*Internal sequencing primers were also used and their sequences are available upon request.

### DNA Sequencing

PCR products were purified by agarose gel electrophoresis by using the MinElute Gel Extraction Kit (QIAGEN) according to the manufacturer’s protocol. Both strands were subjected to direct sequencing by using amplifying primers (Table 2) and additional internal sequence primers. Sequencing reactions were performed by using the ABI PRISM BigDye Terminator version 3.1 Cycle Sequencing Kit (Applied Biosystems, Foster City, CA, USA) according to the manufacturer’s protocol and analysis by using the ABI Prism 3100 system (Applied Biosystems).

### Sequence Analysis

Sequencing data were assembled and analyzed by using the Vector NTI Advance 10 software package (Invitrogen). Nucleotide and deduced amino acid sequences from each isolate were aligned by using the Align X program in Vector NTI and compared with prototype WN-NY99 and previously published sequences of isolates from different regions of the United States and other countries. Phylogenetic relationship studies were based on several methods of analysis (distance, parsimony, and likelihood algorithms) by using MEGA version 3.1 (www.megasoftware.com). All studied isolates were compared with each other, and phylogenetic trees were constructed by using the Kimura 2-parameter method that included transitions and transversions to show genetic relationships of isolates in this study with other WNV isolates in GenBank.

## Results

Because of limited volume of plasma specimens available, viruses were isolated from Vero cell cultures for sequence analysis. We assessed the potential of the viral isolation procedure to cause mutations in the viral genome by comparison of sequences of the structural region of 6 specimens and their respective isolates after 3 passages in Vero cells. There were no changes in the RNA sequences obtained in each original plasma sample and the RNA sequences obtained in each of the passages in culture.

We investigated the genetic variability of 30 WNV isolates collected from plasma specimens from nucleic acid amplification testing–positive blood donors in 13 states during 2002–2005 (Table 1). Isolates were generated by cultivation in Vero cells. The fragment that encompasses the 5′-UTR, all structural genes, and part of *NS1* (bp 1–2,685) from all 30 specimens were subjected to genomic sequencing. Eight of 30 isolates were fully sequenced.

The Appendix Table compares conserved nucleotide mutations and deduced amino acid substitutions identified in structural regions of all 30 isolates with the WN-NY99 isolate (AF196835). Approximately 80% of the nucleotide changes in structural regions were transitions (T ↔ C) and 75% were silent mutations. All mutations in the preM and membrane (M) regions were silent, and 16 isolates shared the transition 660 C → T. Several WNV strains from Europe and Asia, as well as Kunjin virus isolates, also had T at position 660, but both the prototype WN-NY99 and the IS-98 (AF481864) isolates contain 660 C. Twenty-nine of 30 isolates shared 2 conserved nucleotide mutations in the *env* gene: 1442 T → C (Val 159 → Ala) and 2466 C → T that differentiates the new dominant genotype, WN02, from the preceding genotype WN99.

**Figure 1:**
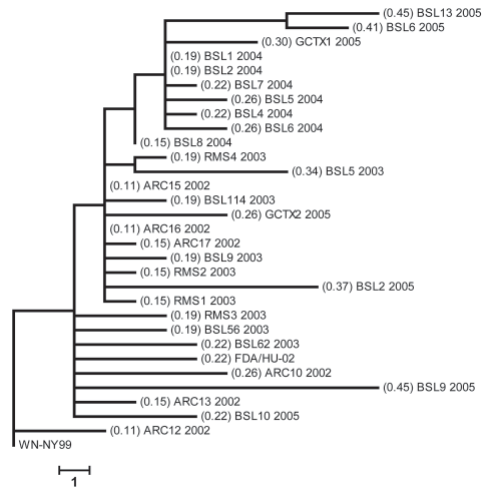
Phylogenetic analyses based on maximum parsimony comparing the 2,685-bp nucleotide sequence, including the complete structural and the 5′-untranslated region of prototype West Nile virus (WNV) strain WN-NY99 with 30 WNV isolates collected during the 2002–2005 epidemics in the United States. Values in parentheses show percentage of nucleotide sequence divergence from WN-NY99. Scale bar represents a 1-nt change.

Construction of a phylogenetic tree by the maximum parsimony method (Figure 1) showed the degree of divergence of isolates from WN-NY99. The average nucleotide divergence for structural genes has increased from 0.18% in 2002 to 0.37% in 2005.

**Figure 2:**
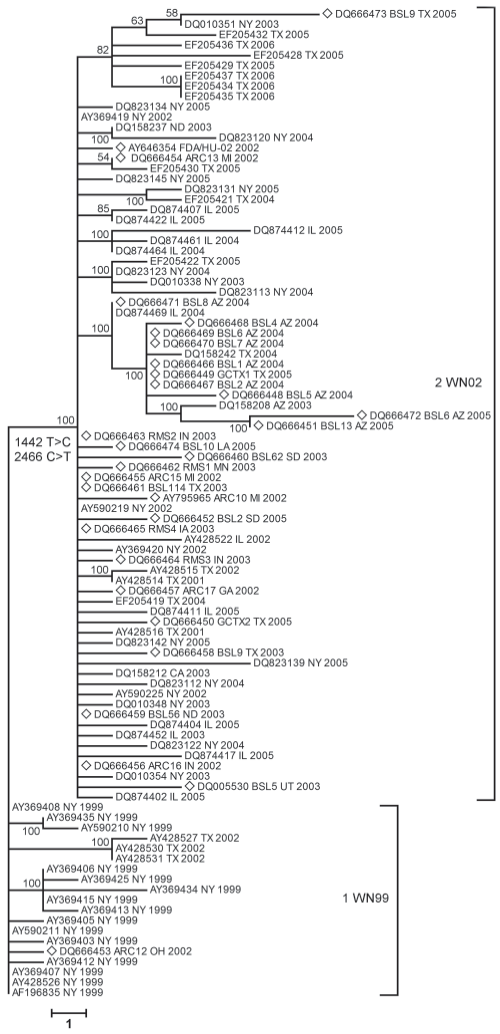
Distance analysis of envelope glycoprotein of West Nile virus isolates collected during 1999–2006 epidemics in the United States. Phylogram is based on maximum parsimony analysis of complete nucleotide sequences of the envelope gene. Diamonds indicate isolates from this study. All isolates from clade 2 (WN02 strain) contained conserved mutations at positions 1442 (T → C) and 2466 (C → T). Values near branches represent percentage support by parsimony bootstrap analysis. Scale bar represents a 1-nt change.

The env protein has several biologic roles, which include viral entry, virion assembly and release, agglutination of erythrocytes, and induction of B- and T-cell responses that are associated with protective immunity. Thus, this protein may be involved in WNV evolution. The phylogenetic tree shown in Figure 2 was constructed by maximum parsimony analysis with *env* gene sequences from US isolates from 1999–2006 in GenBank and sequences of human isolates in our study. The isolates clustered in 2 clades correlated with the parsimony-revealing mutation sites at positions 1442 and 2466.

Although *preM*, *M*, and *env* sequences show adequate phylogenetic representation, we also analyzed 8 complete genomes of WNV isolates for stronger evidence of evolutionary relationships between isolates and additional mutations that may have implications in phenotypic properties of these isolates. Nucleotide changes and deduced amino acid substitutions of complete genomic sequences from 8 isolates are shown in Tables 3 and 4, respectively. When compared with WN-NY99 sequences, these sequences showed an increased number of nucleotide mutations. FDA/Hu-02 isolated in 2002 showed 20 nt mutations plus 1 insertion at position 10497, and 5 mutations resulted in amino acid substitutions on the basis of deduced sequence of viral polyprotein. ARC10–02 isolated in 2002 had 22 mutations, 3 of which resulted in amino acid substitutions. These 2 isolates showed ≈0.2% nucleotide divergence.

**Table 3:** Deduced amino acid substitutions in 8 completely sequenced West Nile virus isolates compared with isolate WN-NY99*

Isolate	Residue no.													
	Nucleocapsid			Envelope glycoprotein									NS1	
	11	21	52	294	298	342	449	472	529	602	663	684	799	895
WN-NY99	Ser	Met	Ala	Leu	Asn	Asn	Val	Glu	Leu	Leu	Ile	Asn	Ile	Leu
FDA/HU-02		Thr	Val				Ala					Ser		
ARC10–02							Ala							
BSL5–03							Ala				Val		Val	
BSL5–04							Ala							
GCTX1–05					Ser		Ala							
GCTX2–05							Ala							Phe
BSL2–05					Ser	Ser	Ala	Gly		Ile				
BSL13–05	Asn			Pro			Ala		Phe					

Protein	NS2A	NS2B	NS3						NS4A					NS4B
Residue no.	1169	1438	1611	1688	1690	1702	1971	1991	2188	2193	2209	2213	2261	2301
WN-NY99	Lys	Ser	Val	Met	Arg	Gly	Pro	Phe	Thr	Phe	Ala	Val	Leu	Ser
FDA/HU-02														Gly
ARC10–02												Ala		
BSL5–03	Arg						Ser							
BSL5–04		Asn							Ser		Thr			
GCTX1–05			Ala	Thr	Lys	Asp				Leu	Thr		Met	
GCTX2–05														
BSL2–05								Leu			Thr			
BSL13–05											Ile			

Isolate	NS4B	NS5												
	2488	2549	2755	2788	2820	2826	2842	3056	3104	3132	3191	3238	3240	
WN-NY99	Ser	Lys	Gly	Asn	Ser	Glu	Lys	Pro	Arg	Arg	Met	Gln	Pro	Total
FDA/HU-02														5
ARC10–02						Gly								3
BSL5–03							Arg							6
BSL5–04			Ser				Arg					Lys	Gln	8
GCTX1–05			Arg	Ser	Leu		Arg		Cys	Ser	Thr			16
GCTX2–05								Ser						3
BSL2–05	Gly	Arg											Gln	10
BSL13–05	Gly						Arg						Gln	8

*NS, nonstructural.

**Table 4:** Nucleotide mutations conserved in fully sequenced West Nile virus isolates from 2002– 2005 epidemics in the United States compared with isolate WN-NY99*

Isolate	Gene or region															
	*preM*	*Env*		*NS2A*	*NS3*				*NS4A*	*NS4B*		*NS5*				*3′-*UTR
	Nucleotide no.															
	660	1442	2466	4146	4803	6138	6238	6426	6721	6996	7015	7938	8621	8811	9352	10851
WN-NY99	C	T	C	A	C	C	C	C	G	C	T	T	A	T	C	A
FDA/HU-02		C	T	G	T	T				T	C	C		C	T	G
ARC10 2002		C	T	G	T	T		T		T	C	C		C	T	G
BSL5 2003	T	C	T	G	T	T		T		T	C	C	G	C	T	G
BSL5 2004	T	C	T	G	T	T	T	T	A	T	C	C	G	C	T	G
GCTX1 2005	T	C	T	G	T	T	T	T	A	T	C	C	G	C	T	G
GCTX2 2005	T	C	T	G	T	T	T	T		T	C	C		C	T	G
BSL2 2005	T	C	T	G	T	T	T	T	A	T	C	C		C	T	G
BSL13 2005	T	C	T	G	T	T	T	T	A	T	C	C	G	C	T	G

*preM, premembrane; Env, envelope; NS, nonstructural; UTR, untranslated region.

The 2003 isolate BSL5–03 showed 39 mutations (nucleotide divergence 0.35%), 7 of which were associated with predicted amino acid substitutions. These 3 isolates from 2002 and 2003 had 11 common mutations: 2 were in env (1442 T → C resulting in Val 449 → Ala and 2466 C → T, a silent mutation); 8 were in the NS regions (4146 A → G in NS2A; 2 C → T transitions at positions 4803 and 6138 in NS3; 6996C → T and 7015T → C in NS4B; T → C transitions at positions 7938 and 8811 and 9352 C → T at NS5); and 1 in the 3-′UTR (10851 A → G).

The 2004 isolate BSL5–04 had 42 mutations with 7 aa substitutions. The isolates from 2005 were as follows: GCTX1-05 had 56 mutations with 17 aa substitutions; GCTX2-05 had 41 mutations with 3 aa substitutions; BSL2–05 had 44 mutations with 10 aa substitutions plus a deletion of 14 nt (10480 to 10493) in the 3′-UTR; and BSL13–05 had 48 mutations with 8 aa substitutions. Isolates from 2004 and 2005 shared a nucleotide mutation 6721 G → A, which resulted in amino acid substitution Ala2209Thr in the NS4A. Four isolates from 3 consecutive years (BSL5–03 from 2003, BSL5–04 from 2004, GCTX1 and BSL13–05 from 2005), shared an amino acid substitution (Lys2842Arg) in NS5. Three isolates from 2005 (GCTX1–05, BSL2–05 and BSL13–05) plus 1 isolate from 2004 (BSL5–04) also shared a silent mutation at position 8550 C → T.

The overall nucleotide divergence from the WN-NY99 isolates from 2003, 2004, and 2005 showed a steady but small increase (BSL5–03, 035%; BSL5–04, 0.38%; BSL2–05, 0.39%; BSL13–05, 0.43%; GCTX1–05, 0.5%; and GCTX2–05, 0.37%). These findings suggest relative stasis in WNV divergence. The 8 completely sequenced isolates shared conserved nucleotide mutations in the *preM*, *M*, *env*, *NS2A*, *NS3*, *NS4B*, and *NS5* genes and the 3′-UTR (Table 4). The largest number of conserved mutations was in the *NS3* and *NS5* genes. No conserved mutations were observed in the *core*, *M*, *NS1*, and *NS2B* genes or the 5′-UTR.

## Discussion

Our study describes genetic variability observed among 30 clinical isolates of WNV from 13 states in the United States obtained during 2002–2005. Since the initial recognition of WNV in North America in 1999 (*10*), the virus has spread from the East to the West Coast and has become endemic. Genetic studies have shown that >50% of all WNV isolates in 2002 and >80% of all isolates in 2003 had a new genotype that emerged in the United States in 2001. The new dominant genotype (WN02) was characterized by 2 conserved nucleotide mutations in *env* (1442 T → C and 2466 C → T) and 1 deduced amino acid substitution (Val 159 → Ala) in the env protein (*16*–*18*). Displacement of the initial genotype (WN99) by WN02 has been attributed to differences in efficiency of transmission of the virus by domestic mosquitoes (*21*). Genetic studies conducted during the early years of WNV activity in the United States identified a small number of mutations and showed no changes suggesting specific adaptations (*6*,*12*,*15*). After the genetic shift in 2001–2002, most nucleotide changes observed were silent transitions (T ↔ C and A ↔ G). Limited variability in structural genes observed by our group and others (*15*,*16*,*18*,*20*,*22*) suggests the absence of strong immune selective pressure, which led to limited evolution of WNV during 2002–2005. Data in this report show that WNV has continued to diverge from precursor isolates as geographic distribution of the virus expanded.

On the basis of nucleotide sequences, 6 aa substitutions were predicted in the core protein of 5 isolates. Two amino acid substitutions, Ser 11 → Asn in isolate BSL13–05 and Met 21 → Thr in isolate FDA-Hu2002, were located within the core hydrophilic region outside α helices. The other amino acid substitutions (Met 34 → Val in isolate BSL06–04, Ala 52 → Val in isolate FDA-Hu2002, Ala 77 → Val in isolate ARC16–02, and Lys 79 → Arg in isolate BSL10–05) were located within α1, α2, α3, and α4 helices, respectively (*23*) and were not expected to affect conformation, function, or antigenic properties of core protein.

Studies of WNV evolution have focused on the env protein because it plays a major role in immune response to infection and immunologic pressure could lead to its variation (*12*,*15*–*21*,*24*,*25*). A total of 29 of 30 isolates in this study contained 2 conserved nucleotide mutations in *env* (1442 T → C and 2466 C → T) that differentiate the dominant genotype from other genotypes and place these isolates in the WN02 clade relative to WN-NY99 (Figure 2). The mutation 2466 C → T was a silent mutation. Mutation 1442 T → C leads to the amino acid substitution Val 159 → Ala, located in the variable region close to the glycosylation site. Glycosylation of env protein can influence virus infectivity and has been considered a potential determinant of virulence in a mouse model (*26*–*28*).

All 7 isolates from 2004 were from Arizona and had 2 common silent mutations in *env*: 1320 A → G present in all isolates, and 1974 C → T, present in 6 of 7 isolates. Mutation 1320 A → G is also present in reported attenuated strains of WNV (AY532665, AY688948, M12294) (*29*) and was also reported in human isolate DQ164201 from Arizona in 2004 and in the red-tailed hawk isolate DQ164204 from Colorado in 2003 (*18*). Mutation 1974 C → T, found in isolates from Arizona and Texas, was detected in 2000 in New York (AF404756), which indicated that this mutation occurred at least twice. A Kunjin virus isolate from 2003 (AY274505) and isolate EthAn4766 from Ethiopia (AY603654) also had that mutation.

**Figure 3:**
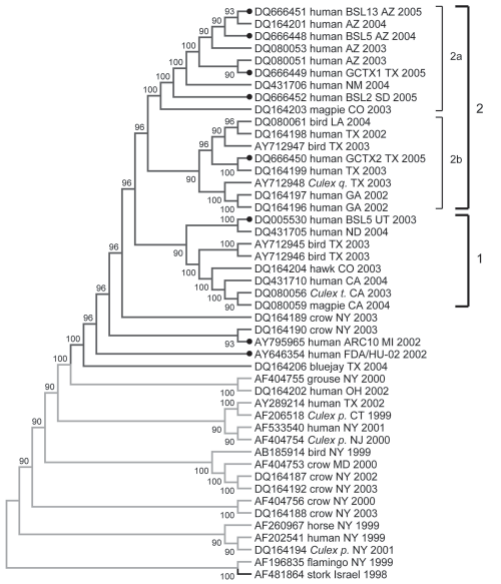
Phylogenetic tree of complete genomes of West Nile virus (WNV) isolates collected during the 1999–2005 epidemics in the United States. Phylogeny reconstruction was estimated by using MEGA version 3.1 (www.megasoftware.com) on the basis of maximum parsimony analysis. Solid circles indicate isolates from this study. Values near branches represent percentage support by parsimony bootstrap analysis. Some parsimony-informative positions (1442, 2446, 4146, 6138, 6721, 8811, 10408, and 10851) play an important role in topologic arrangement of the tree and outgroup configurations. The tree was rooted with prototype WNV isolate WN-NY99 (AF196835) and the most closely related Old World isolate, IS-98 (AF481864). *Culex q*., *Culex. quinquefasciatus*; *Culex t*., *Cx*. *tarsalis*; *Culex p*., *Cx. pipiens*. WNV genotype is color coded: green, WN99; blue, WN02.

Phylogenetic analysis of complete genomes of WNV isolates provided stronger evidence of evolutionary relationships between isolates. Eight fully sequenced isolates were compared by phylogenetic analysis with 39 published complete WNV genomes present in the United States during epidemics in 1999–2005 (Figure 3). The phylogenetic tree was constructed by maximum parsimony analyses and rooted by using WN-NY99 and IS-98; it clearly shows that these 2 isolates were the predecessors of other US WNV isolates. Analysis of nucleotide sequence alignment of complete WNV genomes indicated that most mutations that occur each year were not fixed. However, WNV has continued to diverge and the total number of fixed mutations and the overall nucleotide divergence have increased. Some mutations in US isolates may have appeared as a result of native adaptation and genetic drift of parental WNV isolates (*18*).

Within the WN02 genotype, a sequence from NY 2003 (DQ164189) formed 2 outgroups with moderately strong (96%) bootstrap support (Figure 3). Five of 8 isolates in outgroup 2a were from Arizona and had the 6721 G → A mutation resulting in amino acid substitution Ala 209 → Thr. This mutation was already present in the 2003 magpie isolate DQ164203 from Colorado and in the 2004 human isolate DQ164201 from Arizona (*18*). These isolates also shared silent mutation 8550 C → T, which had been reported in the 2004 human isolate DQ164201 from Arizona (*18*).

Local concentration of closely related isolates in Arizona (Figures 2, 3) or in California (outgroup 1; Figure 3) may have been caused by introduction of 1 or few genetically similar viruses in the area with rapid spread to mosquitoes and local birds, which amplified the original genome. Human infections in that area would therefore result from 1 or a few local WNV-colonizing genotypes and reflect stochastic introduction of 1 or a few infected vectors, followed by rapid localized amplification (*19*). In contrast, a larger number of different viruses were introduced in other areas, as observed in Texas, which lead to a broader diversity of variants.

Several studies showed that mutations in conserved structures within the 3′-UTR did not affect WNV translation but could affect RNA replication and interaction between cellular protein eEF1A and WNV 3′-UTR, facilitating viral minus-strand synthesis (*30*–*32*). Isolate BSL2–05 from South Dakota had a 14-nt deletion (10480–10493) and isolate FDA/Hu-02 had 1-nt insertion at position 10497 in the 3′-UTR; the second isolate had larger plaques than WN-NY99. The recently published 2004 human isolate DQ431705 from North Dakota (*19*) had a 5-nt deletion (10434–10439) in the same 3′-UTR variable region. Previous studies had reported low genetic variation among WNV isolates in North America and emphasized a low frequency of nucleotide variants in the 3′-UTR. Subsequent observations showed that nucleotide changes in the 3′-UTR played a role in rapid spread of this virus in the New World because nucleotide changes in the 3′-UTR had co-evolved with amino acid changes and affected interactions of helicase and RNA polymerase with their RNA substrate (*33*). Mutations in 3′-UTR appear to play a role in production of small-plaque temperature-sensitive variants or mouse-attenuated phenotype variants (*34*).

Our study showed limited but continuous genetic variability with amino acid substitutions in WNV strains. We also found insertions and deletions in the 3′-UTR region that should be studied for assessment of their functional role. Phylogenetic studies of WNV isolates are needed to monitor microevolution of WNV that may affect pathogenic properties of the virus and to assess the effectiveness of available commercial donor screening and diagnostic assays for detection of variant strains. Study of genetic variability may also provide insights into the development of vaccines and therapeutic agents.

## Supplementary Material

Appendix Table Nucleotide changes and amino acid substitutions in structural regions of 30 West Nile virus isolates collected from 2002–2005 epidemics in the United States compared with isolate WN-NY99*
