# Supplementary material for: Genetic Variability of West Nile Virus in US Blood Donors, 2002–2005
Source: Emerg Infect Dis. 2008 Mar;14(3):436–44. doi: 10.3201/eid1403.070463 (PMC2570840; doi:10.3201/eid1403.070463)
Supplement: Appendix Table — Nucleotide changes and amino acid substitutions in structural regions of 30 West Nile virus isolates collected from 2002–2005 epidemics in the United States compared with isolate WN-NY99* [file 07-0463_appT.pdf]

Appendix Table. Nucleotide changes and amino acid substitutions in structural regions of 30 West Nile virus isolates collected from 2002–2005 epidemics in the United States compared with isolate WN-NY99*

Isolate	Gene									
	Core						*prM*	*Env*		
	Nucleotide (amino acid) no.									
	128 (11)	158 (22)	196 (33)	251 (52)	326 (77)	332 (79)	660	977 (294)	989 (298)	1121 (342)
WN-NY99	G (S)	T (M)	A (M)	C (A)	C (A)	A (K)	C	T (L)	A (N)	A (N)
FDA/HU-02		C (T)		T (V)						
ARC10–02										
ARC12–02										
ARC13–02										
ARC15–02							T			
ARC16–02					T (V)		T			
ARC17–02							T			
BSL5–03							T			
BSL9–03							T			
BSL56–03										
BSL62–03										
BSL114–03							T			
RMS1–03							T			
RMS2–03							T			
RMS3–03										
RMS4–03							T			
BSL1–04							T			
BSL2–04							T			
BSL4–04							T			
BSL5–04							T			
BSL6–04			G (V)				T			
BSL7–04							T			
BSL8–04							T			
BSL2–05							T		G (S)	G (S)
BSL6–05							T			
BSL9–05										
BSL10–05						G (R)				
BSL13–05	A (N)						T	C (P)		
GCTX1–05							T		G(S)	
GCTX2–05							T			

Isolate	*Env*									
	1320	1442 (449)	1511 (472)	1652 (519)	1729 (544)	1900 (602)	1974	2083 (662)	2147 (684)	2466 (790)
WN-NY99	A	T (V)	A (E)	G (G)	T (L)	C (L)	C	A (I)	A (N)	C
FDA/HU-02		C (A)							G (S)	T
ARC10–02		C (A)								T
ARC12–02										
ARC13–02		C (A)								T
ARC15–02		C (A)								T
ARC16–02		C (A)								T
ARC17–02		C (A)								T
BSL5–03		C (A)						G (V)		T
BSL9–03		C (A)			C (S)					T
BSL56–03		C (A)								T
BSL62–03		C (A)								T
BSL114–03		C (A)								T
RMS1–03		C (A)								T
RMS2–03		C (A)								T
RMS3–03		C (A)								T
RMS4–03		C (A)								T
BSL1–04	G	C (A)					T			T
BSL2–04	G	C (A)					T			T
BSL4–04	G	C (A)					T			T
BSL5–04	G	C (A)					T			T
BSL6–04	G	C (A)					T			T
BSL7–04	G	C (A)					T			T
BSL8–04	G	C (A)								T
BSL2–05		C (A)	G (G)			A (I)				T
BSL6–05		C (A)		A (E)			T			T
BSL9–05		C (A)					T			T
BSL10–05		C (A)								T
BSL13–05	G	C (A)					T			T
GCTX1–05	G	C (A)					T			T
GCTX2–05		C (A)								T

*prM, premembrane; ENV, envelope. Unique silent mutations are not shown.
